# Perforation of the superior vena cava by a tunnel-cuffed hemodialysis catheter via the right internal jugular vein in an elderly woman

**DOI:** 10.12669/pjms.39.2.6674

**Published:** 2023

**Authors:** Xiaodong Li, Fang Ran, Yancong Guo

**Affiliations:** 1 Xiaodong Li, Department of Nephrology, Baoding No.1 Central Hospital, Baoding, Hebei Province, China; 2 Fang Ran, Department of Nephrology, Baoding No.1 Central Hospital, Baoding, Hebei Province, China; 3 Yancong Guo, Department of Nephrology, Baoding No.1 Central Hospital, Baoding, Hebei Province, China

**Keywords:** Tunnel-cuffed hemodialysis catheter, Internal jugular vein, Superior vena cava

## Abstract

Oliguric patients with acute kidney injury (AKI) often requires an internal jugular vein or femoral venous catheter to establish vascular access for emergency hemodialysis. Puncture with catheterization (PC) of the right internal jugular vein (RIJV) is relatively simple and is often the first choice for hemodialysis catheters insertion. However, complications such as bleeding and hematoma at the puncture site can occur, and in rare cases, the hemodialysis catheter (HDC) can be misplaced into the internal carotid artery, subclavian artery, subclavian vein, or even the thoracic cavity and mediastinum, leading to intractability for processing next. In this study, we report a case of an elderly female patient with AKI who underwent RIJV puncture for long-term HDC because her renal function had not recovered in the short term, and the lower end of the catheter penetrated the superior vena cava (SVC) into the mediastinum due to operator’s carelessness. We did not perform open surgery or endovascular interventions, and instead, the HDC was retained in that place for four weeks and then directly removed without surgery. The patient did not experience any problems, such as bleeding or hematoma, and has been receiving hemodialysis from femoral catheter subsequently since then.

## INTRODUCTION

Acute kidney injury (AKI) is becoming a global public health problem, its incidence rate particularly the number of patients with hemodialysis rises year after year. The main treatment for this disease is renal replacement therapy (RRT), of which kidney transplantation is the first choice and hemodialysis are frequently the second best choice.^1^,^2^ Hemodialysis requires good condition vascular access as a prerequisite. Arteriovenous fistula (AVF) is currently recognized as the best vascular access. but for those patients in whom AVF could not be made. or those who require hemodialysis during AVF maturation, placement of a hemodialysis catheter (HDC) in the central vein is often an appropriate option. However, life-threatening complications may occur with the use of an HDC insertion.^3^,^4^ Currently, there are two general types of HDC: temporary HDC and tunnel-cuffed hemodialysis catheter (TCHDC).

As the number of patients with diabetes and hypertension increases, the number of patients in whom AVF could not be made due to cardiovascular and peripheral vascular disease increases. Therefore, more and more patients undergoing hemodialysis are relying on TCHDC as their primary vascular access.^5^ The internal jugular vein (IJV), particularly the right IJV, is the optimal location for insertion of these catheters. Current guidelines recommend ultrasound-guided the right IJV for catheterization, which is generally a relatively simple and safe procedure with few complications.^6^ However, few vascular injuries during the catheterization of right IJV may lead to the possibility of catheter entry into the mediastinum.^7^ Here, we report an unusual case of a high risk of vascular damage during the catheterization of the right IJV.

In this study, we report a case in which the lower end of the catheter penetrated the superior vena cava (SVC) and went into the mediastinum due to the insertion of right IJV with indwelling TCHDC in an elderly patient.

## CASE DESCRIPTION

An 82-year-old female patient was admitted to the hospital on 08/20/2018. She complained of bilateral lower limb edema for one month and oliguria with chest tightness for three days. She had a 20-year history of hypertension. A month ago, she developed bilateral lower limb edema, which was progressively aggravated, and three days ago, she developed oliguria with chest tightness and came to our hospital. Outpatient examination showed urine protein 3+, ALB 21.12 g/L, CREA 223.82 μmol/L, UREA 19.62 mmol/L, WBC 9.70 ×10^9^/L, HGB 96.00 g/L, and PLT 242.00× 10^9^/L. Then, she was admitted to our hospital with nephrotic syndrome a longed with AKI.

Physical examinations showed severe facial and bilateral lower limb edema. Ultrasound of the kidneys showed echogenic enhancement of both kidney parenchyma. Chest CT showed bilateral pleural effusion, with a large amount on the right side. The results of the main laboratory examinations on admission are shown in Table-I. On the next day, right thoracentesis was performed to drain the chest fluid and on 2018-08-22, a right femoral vein catheterization was performed for continuous hemodialysis. The patient’s chest distress and edema subsided over time, but she still had oliguria.

**Table-I:** Admission Examination.

Investigation	Normal Range	Pre-dialysis	Dialysis after 4 weeks
WBC(×10^9^/L)	4-10	10.8	8.6
Hb(g/L)	110-150	96.5	108.3
Plt(×10^9^/L)	125-350	376	252
Alb(g/L)	40-55	21.2	28.4
24hUpro(g)	0-0.15	5.86	3.78
UREA(mmol/L)	2.60-7.50	18.47	12.46
CREA(umol/L)	41.0-73.0	243.7	165.1
Potassium(mmol/L)	3.50-5.30	5.72	4.53
Calcium(mmol/L)	2.11-2.52	1.98	2.13
Phosphorus(mmol/L	0.85-1.51	1.78	1.32
BNP(pg/ml)	0-100	1240.5	266.3
D-Dimer(mg/L)	0-0.55	3.49	1.86
ESR(mm/h)	0-15	45	22
CRP(mg/L)	0-10	12.5	8.6
Fbg(g/L)	1.8-3.5	5.73	3.84
LVEF(%)	45-55	39	46

WBC (white blood cell), Hb(hemoglobin), Plt(platelet), Alb(albumin), CREA(creatinine), BNP(brainNatriuretic peptide), Fbg(fibrinogen), 24hUpro(24 hours urine proteins), CRP(c reactive protein), LVEF(left ventricular ejection fraction)

**Fig.1:**
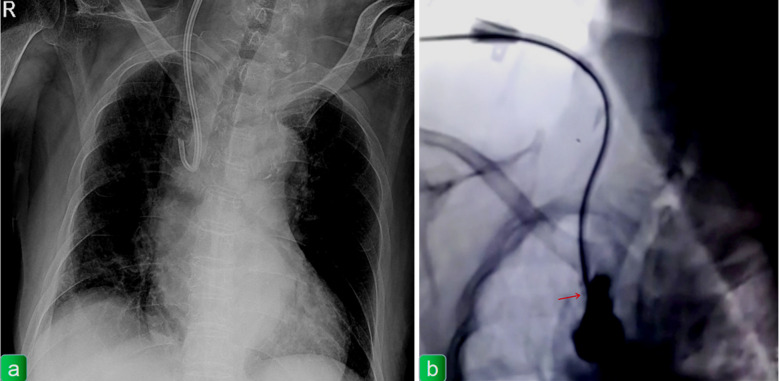
(a) Chest X-ray shows the hemodialysis catheter in the superior vena cava with its front end bent and turning back upward. (b) Digital subtraction angiography (DSA) shows contrast agent was injected into the dual lumen of the dialysis catheter and cumulative in the end of the catheter and did not spread to the periphery. Red arrows indicate the contrast agent accumulation.

**Fig.2:**
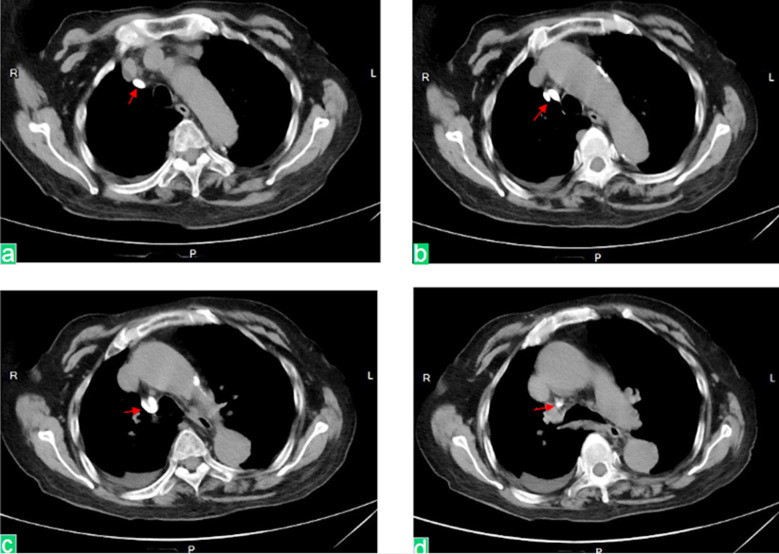
Scan of the chest CT from top to bottom shows the dialysis catheter penetrating the superior vena cava and into the mediastinum. Red arrows indicate the dialysis catheter.

Because she could not survive without hemodialysis, for the time being, she had undergone the insertion of TCHDC through the right IJV on September 18, 2018. The insertion process was performed under ultrasound guidance, and the procedure went successfully. Despite strong resistance from below during the deployment of the guide-wire to the scabbard sheath, the operator forced the insertion and placement of the TCHDC along the sheath. When the guide-wire was removed, the front end of the guide-wire was found bent and deformed, and the dual lumen of the catheter could not be drained out blood with a 5mL syringe. But the patient had no discomfort during the procedure. Images of chest X-ray and chest CT are shown in Fig.1a and 2, respectively. Contrast agent was injected into the dual lumen of the TCHDC and cumulative in the end of the catheter and did not spread to the periphery under digital subtraction angiography (DSA) guidance (Fig.1b).

On urgent consultation with interventional radiologist and vascular surgeons, the TCHDC was retained four weeks before being removed. Her hemodialysis was then maintained by a right femoral vein catheter. After four weeks, the TCHDC was successfully removed under DSA guidance followed by mandatory compressions for 30 minutes. A forearm autologous arteriovenous fistula was established and matured, and the patient has been on maintenance hemodialysis treatment since then in stable condition.

### Patient Consent:

The patient provided her written informed consent for participation in this study.

### Ethics Statement:

The study was approved by Ethics Committee of Baoding the First Central Hospital (Ref: 2020-079).

## DISCUSSION

The most common complication of HDC placement is an infection, especially catheter-related bloodstream infection, followed by venous thrombosis and catheter thrombosis at the catheter site. However, these are late complications, mainly due to prolonged catheter placement.^8^ Compared to the femoral vein, which is often the optimum location for HDC placement and is consequently widely utilized in clinical practice, the right IJV is very straightforward, and the incidence of hemodialysis catheter infection and thrombosis is quite low. The common complication of internal jugular vein PC is local bleeding and hematoma formation at the catheter site, which is relatively simple to manage clinically. However, in a few cases, the catheter can be misplaced into arteries and veins, including the internal carotid artery, subclavian artery, subclavian vein, azygos vein, hepatic vein and even into the mediastinum.^9^ Misplacement of the catheter into the left IJV is the most common, resulting in more difficult postoperative management, which is mostly attributed to the operator’s inexperience and failure to use ultrasound guidance for a puncture.^10^

The patient, in this case, was an elderly patient with nephrotic syndrome with AKI and heart failure, and the TCHDC was retained by the right IJV because her renal function did not recover in the short term and required continued dialysis. The operator was inexperienced, Although the puncture of the right IJV and entrance of guide-wire went successfully under ultrasound guidance, the operator forced the insertion and placement of TCHDC along the sheath despite significant resistance from below during the placement of the guidewire into the sheath. When the guide-wire was removed, the front end of the guide-wire was bent and deformed, and the dual lumen of the catheter could not be drained out blood with a 5 mL syringe.

Imaging further confirmed that the lower end of the hemodialysis catheter penetrated the SVC into the mediastinum, which might be related to the short entry of the guide-wire into the vascular lumen; the end of the guide-wire might not have entered the right atrium or inferior vena cava, and the end of the avulsion sheath might have crossed the end of the guide-wire when it was placed along the guide-wire, thus deviating the placement of the avulsion sheath from the direction of the guide-wire. Furthermore, the operator forced into the sheath when sensing resistance underneath, causing the penetration of guide-wire into the superior vena cava (SVC), and then TCHDC was placed along the sheath, thus causing the lower end of the catheter penetrating the SVC into the mediastinum.

The HDC misplaced into abnormal position of the body should not be removed directly and immediately to avoid hemorrhage, or mediastinal hematoma, compression of the airway, and death.^11^ There are currently three general management options available. First, if there is active bleeding, the broken vessel should be repaired by open surgery performed promptly; however, this procedure is rather traumatic and risky.^12^ Second, the HDC can also be removed under endovascular intervention to repair the vascular rupture. This procedure is generally performed under endoluminal techniques and covered stent-graft, which is less traumatic than open surgery, but require more technical expertise.^13^,^14^ Third, for patients without active bleeding after cannulation, the HDC can also be kept for observation for 2-4 weeks and z`z`zzzDSA-guided after sinus tract formation in peripheral tissue; however, its efficacy is not exact, and endovascular intervention is required if the patient cannot be relieved.^15^ Moreover, the specific method to be used depends on the patient’s condition and the specific location of the HDC.^16^

For the dialysis catheter perforating the SVC, there are usually three methods of choice too.^17^ One is thoracotomy surgery for repair of damaged blood vessels, the other is to perform endovascular intervention or thoracoscopic repair, and the last one is the direct removal of the catheter.^18^-^21^ The exact method varies greatly from person to person, due to the condition of the patient and injured vessels.

In this case, the patient is an elderly woman with long-term hypertension combined with AKI and heart failure, as well as a poor general state that makes open-heart surgery difficult to endure, and the risks of surgery are significant and should be avoided. The TCHDC of the patient penetrated through the SVC into the mediastinum, and no balloon or stent matched the size of the SVC duct to occlude, and endovascular techniques are inappropriate to perform. The TCHDC was kept under observation for four weeks, and on the formation of the peritubular sinus tract, the catheter was successfully removed under DSA guidance followed by mandatory compression. The patient showed no discomfort, no bleeding, no hematoma, and other complications, which could be attributed to her hypercoagulable state due to hypoalbuminemia in nephropathy syndrome. Hence, our case provides a successful experience in conservative therapy rather than surgery for individuals with HDC causing perforation of the SVC and this approach is particularly suitable for patients who are critically ill and cannot tolerate surgery, but its application depends on a comprehensive evaluation of the patient’s condition.

### Limitations:

The limitation of our case was lack of venography of the right IJV after the removal of the TCHDC because of the disagreement of the patient, who thought it was too expensive and unnecessary for her.

## CONCLUSION

To the best of our knowledge, this the first case that reported a TCHDC perforating of SVC via the right IJV in an elderly woman, followed by the manual removal of the catheter successfully. This case is rare in clinical practice. The experience is summarized as follows:

1). It is strongly recommended that preoperative ultrasound should be performed routinely to assess and localize the internal jugular vein diameter and location and to guide the puncture throughout the procedure and that insertion of TCHDC be performed under DSA, if possible, as the operator can visually determine the catheter entry angle, depth, and tip.

2). If HDC is redirected to other areas, avoid removing directly and instead choose for open surgery, endovascular intervention, or removing after four weeks; however, the exact method depends on the patient’s condition and catheter location.

3). Even under ultrasound and DSA guidance, insertion of the right IJV is not always safe to perform. The most important thing is the standard and meticulous operation of the operator, and if resistance is encountered, it is not advisable to force it.Instead, the specific cause should be determined, and active participation of vascular surgery, interventional radiologist is also needed to prevent unexpected complications.^22^

## Data Availability Statement:

The original contributions presented in the case are included in the article, further inquiries can be directed to the corresponding author.

## Authors’ Contributions:

**YG:** Study design, manuscript revision, and responsible and accountable for the integrity of the work.

**XL:** Manuscript writing.

**FR:** Formulating patient’s treatment and data collection.

All authors contributed to the article and approved the submitted version.
